# Methicillin-resistant *Staphylococcus aureus* nasal carriage among janitors working in hospital and non-hospital areas: a comparative cross-sectional study

**DOI:** 10.1186/s12941-020-00391-x

**Published:** 2020-10-19

**Authors:** Seid Abie, Moges Tiruneh, Wondwossen Abebe

**Affiliations:** Department of Medical Microbiology, School of Biomedical and Laboratory Sciences, College of Medicine and Health Sciences, University of Gondar, P.O.Box:196, Gondar, Ethiopia

**Keywords:** MRSA, Nasal carriage, Janitors, Antibiogram profile

## Abstract

**Background:**

Nasal colonization of Methicillin-resistant *Staphylococcus aureus* (MRSA) plays a key role in the epidemiology and pathogenesis of both healthcare-associated and community-acquired MRSA infections in various populations. Screening of MRSA nasal colonization is important in the prevention and control of infection and may provide useful information to guide antimicrobial therapy. This study aimed to determine nasal carriage of MRSA, its antimicrobial susceptibility pattern, and associated factors among janitors working in hospital & non-hospital areas at the University of Gondar, Northwest Ethiopia.

**Methods:**

A comparative cross-sectional study was carried out in a total of 436 study participants (221 hospital and 215 non-hospital janitors) from January to May 2019. The study participants were sampled using a simple random sampling technique. Data on socio-demographic characteristics and associated factors were collected through face to face interviews using a structured questionnaire. Nasal swabs were collected and inoculated into Mannitol salt agar. MRSA was detected using cefoxitin (30 µg) disc and an antibiotic susceptibility test was done using the disc diffusion method. Data were entered and analyzed using SPSS version 20 statistical package. *P* value ≤ 0.05 was considered as statistically significant.

**Results:**

The overall prevalence of *S. aureus* was 101/436 [23.2%, (95% CI: 19.3–27.8)], of which, 29.4% (65/221) were isolated from hospital and 16.7% (36/215) non-hospital janitors. The prevalence of MRSA was 4.8% (21/436) [95% CI: 3.0–6.9]; of these, 8.1% (18/221) of the isolates were from the hospital and 1.4% (3/215) non-hospital janitors, while methicillin-sensitive *S. aureus* (MSSA) in hospital & non-hospital janitors were 49 (22.2%) and 31 (14.4%), respectively. Among the MRSA isolates, 52.4% (11/21) were multi-drug resistant. Of these, 42.9% (9/18) were isolated from hospital and 66.7% (2/3) non-hospital janitors. Hence, nasal carriage of MRSA was significantly associated with hospitalization within the preceding year (AOR = 3.15, CI = 1.13–8.71).

**Conclusion:**

The present study revealed that high MSSA and MRSA were isolated from the hospital as compared to non-hospital janitors and high rates of antibiotics resistance were recorded in the hospital janitors. Consequently, hospitalizations were significantly associated with MRSA. Accordingly, regular screening of carriers in apparently healthy janitors is required for the prevention of nosocomial infections.

## Background

Methicillin-resistant *Staphylococcus aureus* (MRSA) is the most commonly reported multidrug-resistant pathogen in many parts of the world. The rapidly increasing rate of both healthcare-associated MRSA and community-acquired MRSA are major clinical, public health, and economic challenges [1, 2].

Nasal colonization of MRSA plays a key role in the epidemiology and pathogenesis of both healthcare-associated and community-acquired MRSA infection in various populations. It is estimated that up to 7.0% of people in hospitals and up to 2.0% of people in the community are carriers of MRSA infection. MRSA colonization can persist for months to years and the majority of colonized patients remain completely asymptomatic. Besides, MRSA nasal colonization is a risk factor for bacteremia, pneumonia, and skin infection. It is transmitted through person-to-person contact, environmental sources (like doorknobs and handrails), fomites, and companion animals [3–5].

Previous studies in Ethiopia have reported that the rate of nasal carriage of MRSA varying from 0% to 28.9% [6, 7]. A recent meta-analysis study conducted by Reta *et.al* showed that the pooled estimated prevalence of nasal colonization of MRSA was 10.94% [8]**.**

Being a janitor is a predisposing risk factor to a different array of contaminated matter, cleaning agents, wet work, and polluted air & dust. It is also associated with significant increases in respiratory and dermatologic diseases [9]. Besides, screening of MRSA nasal colonization is important in the prevention and control of infection and may provide useful information to guide antimicrobial therapy [3]. However, there are limited data in the study area and Ethiopia in this population [10]. Therefore, this study aimed to determine the prevalence of nasal carriage of MRSA, its antimicrobial susceptibility pattern, and associated factors among janitors working in hospital & non-hospital areas at the University of Gondar, Northwest Ethiopia.

## Methods

### Study area, design, and period

A cross-sectional study was conducted from January to May 2019 among Janitors working in hospital & non-hospital areas at the University of Gondar (UoG). The University of Gondar is one of the oldest and well established higher education institutions located 747 km Northwest of Addis Ababa, the capital city of Ethiopia. It has five campuses namely, College of Medicine and health sciences (CMHS), Maraki, Atse Tewdrose, Atse Fasil, and Tseda, and offers 138 undergraduate and 138 graduate programs. The CMHS has a referral teaching hospital with 700 beds, a maternal and child care hospital, a standard TB ward and laboratory, an eye hospital and Fistula center, and a general hospital that can give all other health services. According to the human resource directorate of the UoG, currently a total of 634 janitors working in the UoG, of which 243 are hospital janitors.

### Sample size and sampling technique

The sample size was determined using Epi-info version 7 in double population proportion formulae. Taking the previous study done at Mekelle University, a 9.7% prevalence of MRSA was used for janitors working in the hospital and 4.9% prevalence of MRSA for janitors working in the non-hospital areas (10). Considering the two prevalence rates, a power of 80% at a 95% confidence level with a 1:1 ratio the sample size was 1002. Since the total number of the source population was less than 10,000, the correction formula was used to adjust and it gives 392. Finally, by considering a 10% non-response rate the final minimum sample size was 436 (221 hospitals and 215 non-hospital janitors). The study participants were sampled using a simple random sampling technique. In brief, a compressive list of janitors was obtained from the UoG human resource directorate office and the list was stratified into a hospital and non-hospital janitors. The selection was performed using simple random sampling by lottery methods until we get a preferred sample in each group.

### Data collection

Data were collected through face to face interview using a structured questionnaire containing information on basic demographic characteristics and possible risk factors for colonization of MRSA such as age, gender, education level, job durations, workplace, use of antibiotics, recent respiratory and skin infections, presence of wounds and allergies, recent hospitalization, having a child, exposure to infectious waste and living with the hospitalized person.

### Specimen collection, processing, and identification

A nasal swab was collected from each anterior nare using a sterile moistened swab. Each swab was rubbed five times against the anterior 1 cm of the nasal vestibular wall of both nares and immediately placed into Tryptose soya broth (Oxoid Ltd., Basingstoke, UK). The specimens were transported to the University of Gondar Medical Microbiology Laboratory in a cold box with ice-packs within 1 h of collection for further processing. The swabs were directly inoculated onto Mannitol salt agar (Oxoid Ltd. England), incubated at 37 °C for 48 h, and left at room temperature to stimulate pigment formation. The isolates were characterized as *S.aureus* based on morphology, Gram stain, catalase test, coagulase test, and mannitol salt agar fermentation [10].

### Antimicrobial susceptibility test

Antimicrobial susceptibility testing was performed on Muller Hinton Agar (Oxoid, UK) using the disc diffusion method against penicillin G (10 units), gentamicin (10 µg), erythromycin (15 µg), tetracycline (30 µg), trimethoprim/sulphamethoxazole (25 µg), ciprofloxacin (5 µg), clindamycin (15 µg) and cefoxitin (30 µg) based on the guidelines adapted from Clinical and Laboratory Standards Institute (CLSI 2019 edition); the results were reported as sensitive, intermediate and resistance as previously described by CLSI [11]. Besides, MRSA strains were differentiated from methicillin-sensitive *S.aureus* (MSSA) strains using cefoxitin (30 µg) disc, *S.aureus* with a zone of inhibition of $\le$ 21 mm was considered to be MRSA phenotypically [11]. Multidrug resistance (MDR) is defined as the resistance of an isolate to three and more antimicrobial classes [12]. *S. aureus* American Type Culture Collection (ATCC) 43300 (MRSA) and ATCC 25923 (MSSA) were used as the quality control strain [11].

### Data analysis and interpretation

Data were entered and analyzed using SPSS version 20 software. Descriptive statistics were employed and binary logistic regression analysis was used to check the association between dependent and independent variables with odds ratio at 95% confidence intervals. All independent variables with p-value $\le$ 0.2 in the bivariate analysis were included in a multivariate logistic regression model. P-value $\le$ 0.05 in multivariate analysis was considered as statistical significance.

## Results

### Characteristics of the Study participants

A total of 436 study participants (221 hospital and 215 non-hospital janitors) were included in this study. The majority of the janitors were females, 420 (96.3%) and their ages ranged from 18 to 58 years with a mean age of 28.15 ± 6.3 years. Three hundred one (69%) of the participants had attended college & above and 259 (59.4%) were married. Most of the janitors had served less than 2 years (52.1%). Hundred eighty-five (42.4%) suffered from allergic rhinitis and 221 (52.0%) had exposure to waste & body fluids. However, 173 (39.7%) participants suffered from respiratory tract infection in the last 3 months, 128 (29.4%) had skin infections, 170 (40%) were prescribed antibiotics for the last 3 months, 119 (27.3%) were currently living with children, and 105 (24.9%) lived with a hospitalized person (Table 1).

**Table 1:** Descriptive characteristic of janitors working in the hospital and non-hospital areas at the UoG, Northwest Ethiopia, 2019

Variables	Workplace		Total (n = 436)
	Hospital (n = 221)	Non-hospital (n = 215)	
Sex			
Male	12 (5.4)	4 (1.9)	16 (3.7)
Female	209 (94.6)	211 (98.1)	420 (96.3)
Mean (SD) Age (range)	30.22 ± 7.8 (18–58)	26.0 ± 3.17 (20–39)	28.15 ± 6.3 (18–58)
Age group(years)			
18–25	65 (29.4)	102 (47.4)	167 (33.3)
> 25	102 (47.4)	113 (52.6)	269 (61.7)
Marital status			
Single	91 (41.2)	56 (26)	147 (33.7)
Married	121 (54.8)	138 (64.2)	259 (59.4)
Divorced	9 (4.1)	21 (9.8)	30 (6.9)
Educational status			
No formal education	7 (3.2)	0	7 (1.6)
Secondary school	103 (46.6)	25 (16.6)	128 (29.4)
College and above	111 (50.2)	190 (88.4)	301 (69)
Currently living with children			
Yes	64 (28.9)	55 (25.6)	119 (27.3)
No	157 (71.1)	160 (74.4)	317 (72.7)
Prescribed antibiotics in the last 3 months			
Yes	105 (47.5)	65 (44.2)	170 (40)
No	116 (52.5)	150 (55.8)	266 (60)
Respiratory tract infection in the last 3 months			
Yes	98 (44.3)	55 (39.5)	153 (35.1)
No	123 (55.7)	160 (60.5)	283 (64.9)
Allergies			
Yes	67 (30.3)	118 (54.9)	185 (42.4)
No	154 (69.7)	97 (45.1)	251 (57.6)
Skin infection in the last 3 months			
Yes	98 (44.3)	30 (14)	128 (29.4)
No	123 (55.7)	185 (86)	308 (70.6)
Wound infection in the last 3 months			
Yes	50 (22.6)	38 (17.7)	88 (20.2)
No	171 (77.4)	177 (82.3)	348 (79.8)
Exposed to body fluids			
Yes	83 (37.6)	128 (59.5)	221 (50.7)
No	128 (62.4)	87 (40.5)	215 (49.3)
Hospital stays in the last 3 months			
Yes	50 (22.6)	12 (5.6)	62 (14.2)
No	171 (77.4)	203 (94.4)	374 (85.8)
Living with a hospitalized person			
Yes	67 (30.3)	38 (17.7)	105 (24.1)
No	154 (69.7)	177 (82.3)	331 (75.1)
Total job duration as a janitor			
< 2 years	125 (56.6)	102 (47.4)	227 (52.1)
≥ 2 years	96 (43.4)	113 (52.6)	209 (47.9)

### Nasal carriage of MSSA and MRSA among hospital and non-hospital janitors

The nasal carriage rate of *S. aureus* was 23.2% (101/436). Of these, 29.4% (65/221) were isolated from hospital and 16.7% (36/215) non-hospital janitors. There was a statistically significant difference in the isolation frequency of *S. aureus* among hospital and non-hospital janitors (*p* = 0.013). The prevalence of MSSA and MRSA was 18.3% (80/436) and 4.8% (21/436), respectively. Among 21 MRSA isolates, 8.1% (18/221) were from the hospital and 1.4% (3/215) non-hospital janitors, while MSSA in hospital & non-hospital janitors were 49 (22.2%) and 31 (14.4%), respectively. There was no significant association between the carriage rate of MRSA/MSSA and the workplace (*p* = 0.092) (Table 2).

**Table 2:** Nasal carriage of *S. aureus* and MRSA among hospital and non-hospital janitors working at the UoG, Northwest Ethiopia, 2019

	Hospital janitors (n, %)	Non-hospital janitors (n, %)	Total (n, %)	OR (95% CI)	*P* value
Number of participants	221	215	436		
*S.aureus*	65 (29.4)	36 (16.7)	101 (23.2)	2.127 (1.170–3.866)	0.013
MSSA	49 (22.2)	31 (14.4)	80 (18.3)	1.490 (0.912–2.435)	0.112
MRSA	18 (8.1)	3 (1.4)	21 (4.8)	3.294 (0.822–13.195)	0.092

*MRSA* Methicillin resistant *S.aureus, MSSA* Methicillin susceptible *S.aureus*

### Antimicrobial susceptibility testing

Table 3 presents antibiotic resistance patterns of 101 *S.aureus* (MRSA & MSSA) strains isolated from 436 study participants. Overall, 31.7% (32/101) isolates were resistant to three or more classes of antibiotics tested (MDR). Of these, 20.8% (21/101) were MSSA and 10.9% (11/101) were MRSA. Among the total of 80 MSSA isolates, 26.3% (21/80) were MDR. Of these, 36.7% (18/49) were isolated from nasal nares of the hospital and 9.7% (3/31) non-hospital janitors. Among the total of 21 MRSA isolates, 52.4% (11/21) were MDR. Of these, 42.9% (9/18) were isolated from nasal nares of the hospital and 66.7% (2/3) non-hospital janitors. MRSA isolates were resistance to penicillin 21(100%), trimethoprim/sulphamethoxazole 9(42.9%), ciprofloxacin 8(38.1%), tetracycline 7(33.3%), erythromycin 5(23.8%), gentamicin 5(23.8%), and clindamycin 4(19%). MSSA isolates were resistance to penicillin 78(97.5%), trimethoprim/sulphamethoxazole 13(30.0%), ciprofloxacin 7(8.8%), tetracycline 16(20%), erythromycin 17 (21.3%), gentamicin 16.3% and clindamycin 2(2.5%). Accordingly, there was a statistically significant difference of resistance to tetracycline, gentamicin, ciprofloxacin, and clindamycin between the hospital and non-hospital isolates (*p* < 0.05).

**Table 3:** Antibiogram profile of MRSA & MSSA, isolated from janitors working in the hospital and non-hospital areas at the UoG, Northwest Ethiopia, 2019

Antibiogram pattern	Nasal carriage of MRSA (n = 21)		MDR pattern of MRSA	Antibiogram pattern	Nasal carriage of MSSA (n = 80)		MDR pattern of MSSA
	HJ (n = 18)	N-HJ (n = 3)			HJ (n = 49)	N-HJ (n = 31)	
PEN	0	0	0	PEN	20	19	39
PEN & ERY	2	0	2	TET	1	0	1
PEN & CLI	2	0	2	ERY	0	1	1
PEN & SXT	2	1	3	PEN & ERY	5	1	6
PEN & CIP	3	0	3	PEN & GEN	3	2	5
PEN,GEN & SXT	1	1	2	PEN & SXT	1	4	5
PEN, TET & CLI	1	0	1	PEN & CLI	1	0	1
PEN, TET& SXT	1	0	1	PEN & TET	0	1	1
PEN, CIP& SXT	0	1	1	PEN, SXT & TET	2	0	2
PEN, CIP,SXT & ERY	1	0	1	PEN, GEN & CIP	1	0	1
PEN, TET, CLI & SXT	1	0	1	PEN, GEN & TET	2	0	2
PEN, GEN,CLI, SXT & ERY	1	0	1	PEN, TET & ERY	3	0	3
PEN, GEN,CIP,TET & ERY	1	0	1	PEN,SXT & ERY	2	0	2
PEN,GEN,CIP, TET & SXT	2	0	2	PEN, GEN, CIP & TET	2	1	3
				PEN, GEN, TET & SXT	1	1	2
				PEN, GEN,SXT & ERY	1	1	2
				PEN, GEN, CLI, TET & ERY	1	0	1
				PEN, GEN, CIP, TET & ERY	1	0	1
				PEN, GEN, CIP, SXT & ERY	2	0	2

*HJ* hospital janitors, *N-HJ* non-hospital janitors, *MRSA* Methicillin resistant *S.aureus*, *MSSA* Methicillin susceptible *S.aureus,* *PEN* Penicillin, *ERY* Erythromycin, *CIP* Ciprofloxacin, *TET* Tetracycline, *CLI* Clindamycin, *SXT* Trimethoprim/sulphamethoxazole, *GEN* Gentamicin

### Factor associated with nasal carriage of *S.aureus* and MRSA

In multivariate logistic analysis, associations were observed between nasal carriage of *S.aureus* and work place (AOR = 2.12, 95% CI = 1.17–3.86), respiratory tract infection (AOR = 2.37, 95% CI = 1.02–5.46), skin infection (AOR = 6.37, 95% CI = 3.61–11.77), wound infection (AOR = 2.57, 95% CI = 1.38–4.77) and living with hospitalized person (AOR = 3.15, 95% CI = 1.67–5.94). However, nasal carriage of MRSA was significantly associated with hospitalization within the preceding year (AOR = 3.15, CI = 1.13–8.71) (Table 4).

**Table 4:** Multivariate analysis of factors associated with *S. aureus* and MRSA nasal carriage among janitors working at the UoG, 2019

Variables	Nasal carriage of *S.aureus* (n = 101) (n,%)	AOR (95% CI)	*P* value	Nasal carriage rate of MRSA (n = 21) (n,%)	AOR (95% CI)	*P* value
Age group (years)						
18–25	32 (31.7)		0.984	8 (38.1)	–	
> 25	69 (68.3)	1.006 (0.580–1.745)		13 (61.9)		
Sex						
Male	4 (4)		–	0	–	
Female	97 (96)			21 (100)		
Marital status						
Single	30 (29.7)	–		4 (19)	–	
Married	66 (65.3)			15 (71.4)		
Divorced	5 (4.9)			2 (9.5)		
Educational status						
Secondary school or less	42 (41.6)	–	–	11 (52.4)	1.178 (0.481–3.221)	0.750
Collage or above	59 (58.4)			10 (47.6)		
Currently living with children						
Yes	27 (26.7)	–	–	5 (23.8)		–
No	74 (73.3)			16 (76.2)		
Prescribed antibiotics in the last 3 months						
Yes	47 (46.5)	–	–	11 (52.4)		
No	54 (53.5)			10 (47.6)		
Respiratory tract infection in the last 3 months						
Yes	13 (12.9)	2.371 (1.029–5.463)	0.043	3 (14.3)		
No	88 (87.1)			18 (85.7)		
Allergies						
Yes	37 (36.6)	0.871 (0.508–1.494)	0.616	6 (28.6)	0.796 (0.286–2.217)	0.662
No	64 (63.4)			15 (71.4)		
Skin infection in the last 3 months						
Yes	46 (45.5)	6.378 (3.617–11.248)	0.000	5 (23.8)		
No	55 (54.5)			16 (76.2)		
Wound infection in the last 3 months						
Yes	32 (31.7)	2.570 (1.383–4.774)	0.003	2 (9.5)		
No	69 (68.3)			19 (90.5)		
Exposed to body fluids						
Yes	55 (54.5)	1.352 (0.395–2.298)	0.266	10 (47.6)		
No	46 (45.5)			11 (52.4)		
Hospital stays in the last 3 months						
Yes	21 (20.8)	1.292 (0.643–2.595		9 (42.9)	3.151 (1.139–8.714)	0.027
No	80 (79.2)			12 (57.1)		
Living with a hospitalized person						
Yes	36 (35.6)	3.151 (1.670–5.945)		8 (38.1)	1.551 (0.531–4.528)	0.442
No	65 (64.4)			13 (61.9)		
Job duration as a janitors						
< 2 years	56 (55.4)	–		11 (52.4)	0.597 (0.215–1.655)	0.321
≥ 2 years	45 (44.6)			10 (47.6)		
Workplace						
Hospital	65 (64.4)	2.127 (1.170–3.866)	0.013	18 (85.7)	3.294 (0.822–13.195)	0.092
Non-hospital	36 (35.6)			3 (14.3)		

*MRSA* Methicillin-resistant *S.aureus*

## Discussion

The present study reveals information on the nasal carriage of *S. aureus* and MRSA along with their antibiotic susceptibility pattern and factor associated among janitors working in the hospitals and non-hospital areas that play a decisive role to prevent future infection. The prevalence of nasal carriage *S. aureus* in this study was 23.2%. This was consistent with those studies conducted among medical students in Jimma, Ethiopia (22.1%) [12], and the poor urban community of San Francisco, USA (22.8%) [13]. However, it was higher than the results of studies reported among janitors working in the hospital and non-hospital areas in Mekelle, Ethiopia (17.9%) [10] and Taiwan (15.3%) [14]. The overall prevalence of nasal carriage of MRSA in this study (4.8%) was in agreement with a study conducted in Mekelle, Ethiopia (6.25%) [10]; on the contrary, this finding was lower than the results of the studies conducted in Dessie, Ethiopia (12.7%) [15], Jimma, Ethiopia (8.4%) [12] and Gaza strips, Palestine (22.6%) [16]. But, higher than the results of the studies conducted in Taiwan (2.7%) [14] and Madagascar (1.3%) [17]. The difference might be due to variation in study population, sample size, laboratory methods, prevention, and infectious control policies across/within countries and the degree of exposures to the risky environment.

The prevalence of *S.aureus* was higher in hospitals (29.4%) than non-hospital janitors (16.7%); this was statistically significant *(p* = 0.013). This result was in line with those of the studies conducted in Mekelle (hospital janitors = 25.2% vs. non-hospital janitors = 15.3%) [10] and Taiwan (hospital janitors = 15.3% vs. non-hospital janitors = 13.3%) [14]. Moreover, the nasal carriage rate of MRSA among janitors working in hospitals (8.1%) was higher than among those working in non-hospital janitors (1.4%) (But it was not statistically significant). This was in agreement with a study conducted in the USA [healthy care workers (7%) vs. non-healthcare workers (2%)] [18]. The higher carriage rate in hospital janitors might be due to high rates of skin infections, wound infections, respiratory infections, frequent use of broad-spectrum antibiotics, high chance of exposure to contaminants & higher selective pressure in the hospital area. However, Most of the janitors working in the hospitals did not follow the correct procedures of medical waste disposal and hand hygiene due to lack of personal protective equipment in the present study. Those asymptomatic colonized individuals have been risky to the hospital or the community because the janitors keep on working unaware of the colonized (unless clinical infection develops), he/she is contributing to the spread of MRSA.

All MRSA isolates were 100% resistant to penicillin, while the MSSA isolates were 97.5% resistant to penicillin. Isolates of MRSA and MSSA from hospital janitors were more resistant to the different classes of antimicrobials than isolates from non-hospital janitors. However, the resistance pattern of MRSA isolates to tetracycline, gentamicin, ciprofloxacin, and clindamycin were significantly higher in-hospital than non-hospital janitors. About 30.7% of the isolates were MDR and a high number of MRSA isolates were multidrug-resistant as compared with the MSSA isolates. This might be due to that, the hospital janitors had higher chances of exposure to infectious patients and contaminated or disposable waste materials.

In the present study, skin infection, wound infection, respiratory infection in the last 3 months, living with a hospitalized person, and working area was found to be more significantly associated with *S.aureus* than MRSA. Besides, hospitalization within the last 3 months has been found significantly associated with MRSA. This confirms that nasal carriage of *S.aureus* has been associated with an increased risk for skin infection, wound infection, and respiratory infection. The high colonization rate among hospital janitors might be since hospital janitors had a higher chance of exposure to contaminated and disposable waste materials.

## Limitation of the study

The study has some limitations, We were unable to perform vancomycin minimum inhibitory concentration and use advanced molecular techniques due to budget constraint.

## Conclusion

The present study revealed that high MSSA and MRSA isolates were isolated in hospitals as compared to non-hospital janitors and a large number of resistances to the tested antibiotics were found in the hospital isolates. Moreover, hospitalizations were significantly associated with MRSA. Accordingly, we advocate regular screening of carriers on apparently healthy cleaners is required for the prevention of nosocomial infections.

## Data Availability

All data generated or analyzed during this study were included in this article.

## Abbreviations

MDR: Multiple Drug Resistance
MRSA: Methicillin resistance *Staphylococcus aureus*
MSSA: Methicillin-susceptible *Staphylococcus aureus*
UoG: University of Gondar
